# Transcutaneous electrical nerve stimulation with the injection of pethidine and promethazine in the labor pain reduction: A randomized controlled trial

**DOI:** 10.22088/cjim.14.4.628

**Published:** 2023

**Authors:** Nafiseh Saedi, Nasim Shokouhi, Elham Feizabad, Zahra Moghimi, Mona Mohseni

**Affiliations:** 1 Department of Obstetrics and Gynecology, Yas Hospital, Tehran University of Medical Sciences, Tehran, Iran

**Keywords:** Analgesia, Obstetrical. Analgesia, Patient-Controlled, Labor Pain, Pain Management, TENS.

## Abstract

**Background:**

The use of transcutaneous electrical nerve stimulation (TENS) to relieve labor pain remains controversial and existing evidence is neither strong nor consistent. This research was designed to compare TENS' effect with the injection of pethidine and promethazine in labor pain reduction.

**Methods:**

In this trial, for 45 pregnant women in the active phase of labor, TENS electrodes were placed (two on both arms, and two over the participants’ low back) continuously for 120 minutes; and for another group 45 pregnant women, 100 milligrams of pethidine and 250 micrograms of promethazine were injected intramuscularly which could be repeated once at least one hour later. Labor pain and duration, need for labor induction/augmentation/other pain control methods/ instrumental delivery, delivery type, and maternal and newborn complications were measured in both groups.

**Results:**

The baseline mean visual analog scale (VAS) score, in the TENS group was 8.51±0.62 and in the pethidine and promethazine groups was 8.37±0.61 (P=0.31). While in a 120min post-intervention, it was 6.29±1.50 and 5.73±1.46 in the TENS group and the pethidine and promethazine group, respectively with no statistically significant difference (P=0.07). The labor duration in the TENS group was 6.61±1.71 hours and in the pethidine and promethazine group was 6.17±2.07 hours, with no statistically significant difference (P=0.33). In addition, no complication was recorded neither in the mothers nor newborns.

**Conclusion:**

This study showed that applying TENS in the active labor phase can reduce at least two scores in patient labor pain with no significant complications.

Labor pain, is an unavoidable part of a normal vaginal delivery (NVD). Notably, women who experience high levels of pain during their NVD are at higher risk for subsequent complications such as vaginal and/or anal sphincter injuries, fetal tachycardia, and fetal blood sample abnormality (1, 2). Considering the importance of labor pain relief, the efficacy of various intrapartum analgesia methods has been evaluated (3, 4). Overall, there are two approaches for labor pain management: pharmacologic and non-pharmacologic. Of them, opioid agents and epidural analgesia (EA) are the most commonly used techniques (5).

Although these methods are used widely, there are important concerns about their side effects. For instance, pregnant women who experienced EA are more susceptible to having long-lasting first and second labor stages, maternal hypotension, motor neuron block, fever, urinary retention, and needing augmentation or induction by oxytocin as well as increasing risk of instrumental delivery (6, 7).

In contrast, evidence showed that non-pharmacological pain relief approaches could be safe, non-invasive, inexpensive, and easily applicable. Furthermore, these approaches decline analgesic agent prescription during labor and constantly their adverse effects (8-10). One of the non-pharmacological methods nowadays used, is the transcutaneous electrical nerve stimulation (TENS). The role of TENS in pain reduction is through large-diameter afferent fiber activation. These afferent fibers' input is sent to the central nervous system to activate descending inhibitory system pathways which result in reduce labor pain (11). Furthermore, in TENS method, the parturient can control both the frequency and intensity of the low-voltage electrical impulses emitted from the TENS device through electrodes applied to the lower back. Despite these advantages, our knowledge about any negative effects of it on both the mother and the fetus is insufficient (12-15).

As mentioned, the application of TENS to reduce labor pain still remains controversial and existing evidence is neither strong nor consistent. On the other hand, there is no guarantee of the full effectiveness of existing painless approaches, hence, evaluation and replacement of new interventions especially noninvasive and non-pharmacological strategies such as TENS seem needed. Hence, this research was designed to compare TENS' effect with the injection of pethidine and promethazine in labor pain reduction.

## Methods

The study was conducted in compliance with the Helsinki Declaration. It was confirmed by Tehran University of Medical Sciences Ethics Committee (IR.TUMS.MEDICINE.REC.1398.013) and the IRCT registry number was IRCT20190208042655N1. All the pregnant women signed the informed consent prior to enrolling in the study. This non-blinding randomized controlled study was performed on 90 pregnant women (45 in TENS intervention group and 45 in pethidine and promethazine intervention group) in Yas Hospital, Tehran, Iran, 2021. The study included term (gestational age: 37 to 41 weeks) pregnant women at age of 16 to 40 years, with singleton pregnancy, cephalic presentation, and spontaneous vaginal delivery. Pregnant women with preeclampsia, gestational diabetes mellitus managed with insulin, intrauterine growth restriction, meconium staining, and history of previous cesarean section or previous transmural myomectomy or uterus rupture were excluded.

A random allocation rule was used to assign participants to the study intervention groups; TENS and pethidine and promethazine groups. The study was non-blinded because of the nature of the interventions.

In the first group of intervention, at the active phase of labor (cervical dilation: 4 cm with uterine contractions), two pairs of TENS electrodes were placed on participants’ both arms, and two electrodes over the participants’ low back. A TENS system (Body Clock Health Care Company, England) was set at 15 milliampere and 300 volts, continuously for 120 minutes. In the second group of intervention, 100 milligrams of pethidine (EXIR Pharmaceutical Company, Iran) and 250 micrograms of promethazine (EXIR Pharmaceutical Company, Iran) were injected intramuscularly at active phase of labor, if the pregnant woman was not responding to the primary dose; the second dose was applied at least one hour later.

All pregnant women received routine obstetric care. Labor pain was measured by visual analog scale (VAS), from 0 (no pain) to 10 (worst imaginable pain) at baseline and 120min post-intervention. The secondary study outcomes were labor duration, the need for labor induction and augmentation, the need for other pain control methods, the need for instrumental delivery, delivery type, maternal complications, fifth minute neonatal Apgar, umbilical arterial pH, and the need to neonatal intensive care unit (NICU) admission. 

All data were analyzed with SPSS Version 24.0. A p-value of less than 0.05 was considered as the level of statistical significance. For continuous variables, mean ± standard deviation and for qualitative variables, frequency and percentage were used. In addition, the chi-square test was used to assess the differences in proportion. 

## Results

**Figure 1:**
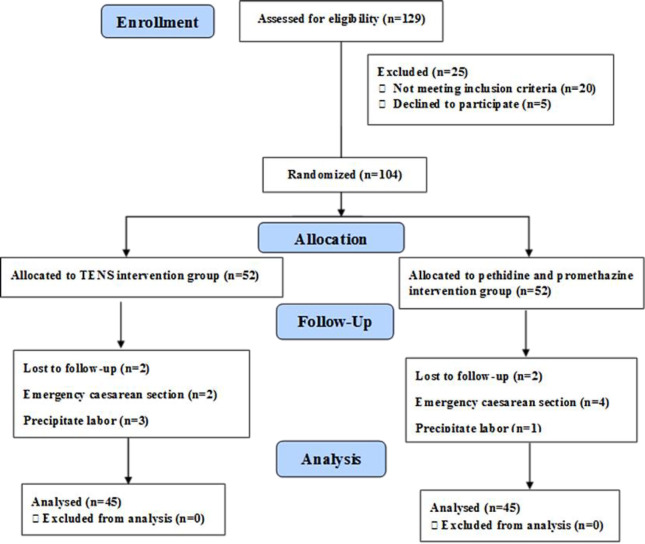
The study flow diagram

We assessed a total number of 129 pregnant women, 25 of them were excluded from the study (20 women due to not meeting the inclusion criteria and 5 women declined to participate). Then, 52 women were randomly assigned into TENS intervention group and 52 women into pethidine and promethazine intervention group. During the study, 14 women (7 from each group) left the study due to different causes. Finally, the study analysis was done on 90 pregnant women (figure 1). The baseline information of the study groups including maternal age, BMI, gravidity, parity, gestational age, and bishop score did not differ significantly (table 1). Before the study interventions, the mean VAS score in TENS group was 8.51±0.62 and in pethidine and promethazine group was 8.37±0.61, with no statistically significant variation (p>0.05, table 2). In a 120min post-intervention, the VAS score average was 6.29±1.50 and 5.73±1.46 in TENS group and pethidine and promethazine groups, respectively with no statistically significant difference (p>0.05, table 2). The labor duration in TENS group was 6.61±1.71 hours and in pethidine and promethazine group was 6.17±2.07 hours, no statistically significant difference existed in terms of the labor duration (p>0.05, table 2). There was no statistically significant variations among the groups in terms of the other sedation drug use, augmentation, instrumental delivery, cesarean section, fifth minute neonatal Apgar, umbilical cord PH, and NICU admission among the two groups (p>0.05, table 3).

**Table 1:** The participant baseline information

**Characteristic**	TENS* **group (N =45)**	**Pethidine and Promethazine group (N =45)**	**P-value**
Maternal age, yrs.	31.84±2.50	30.56±4.86	0.119
Body mass index, Kg/m^2^	27.77±5.19	27.6±5.04	0.869
Gravidity, n	1.53±0.50	1.55±0.75	0.870
Parity, n	0.42±0.49	0.40±0.49	0.833
Gestation age, weeks	39.71±1.07	39.37±1.00	0.133
Bishop score, n	1.71±0.45	1.64±0.48	0.504

TENS: Transcutaneous electrical nerve stimulation.

**Table 2:** Comparison of labor pain information in two groups

**Characteristic**	**TENS** **group (N =45)**	**Pethidine and Promethazine ** **group (N =45)**	**P-value**
Duration of labor, hours	6.61±1.71	6.17±2.07	0.332
Pain before the intervention	8.51±0.62	8.37±0.61	0.310
Pain 120min after the intervention	6.29±1.50	5.73±1.46	0.079

TENS: Transcutaneous electrical nerve stimulation.

**Table 3:** Comparison of delivery and newborn information in two groups

**Characteristic**	**TENS** **group (N =45)**	**Pethidine and Promethazine** **group (N =45)**	**P-value**
Other sedation drug use	10 (22.2)	8 (17.8)	0.396
Augmentation	42 (93.3)	38 (84.4)	0.157
Instrumental delivery	1 (2.2)	1 (2.2)	0.753
Cesarean section	10 (22.2)	7 (15.6)	0.296
Apgar score less than 7	2 (4.4)	1 (2.2)	0.500
Umbilical cord PH less than 7.1	2 (4.4)	0	0.247
Neonatal intensive care unit admission	2 (4.4)	0	0.247

TENS: Transcutaneous electrical nerve stimulation.

## Discussion

This study has shown that the use of TENS at the first time of active phase can be reduced at least two scores of VAS in labor pain measuring. In similar, recent studies (13, 14, 16) have shown a small, but statistically significant efficacy of TENS on the reduction of labor pain intensity that may even last until four hours after labor (17).

Njogu et al. trial (18) showed that TENS group had significant (p< 0.001) less mean VAS scores at a different labor time until 24 h after delivery and a significant (p< 0.001) shorter duration of the active labor compared to the control group. While our findings did not demonstrate a significant difference regards labor pain and active labor phase length; it might be due to having two intervention groups, and for the other study groups, pethidine and promethazine were injected and high technology Bio-feedback TENS system use in Njogu et al. trial. TENS usage at the beginning of the active phase of labor causes decrease in labor pain even in pregnant women having fetus with breech presentation (19).

 In addition, Santana et al. (20) study showed that in TENS group the meantime for the women who requested for neuraxial labor analgesia was longer compared to the control group, although we did not evaluate this variable. Similar to other previous studies, no maternal and neonatal negative impacts were reported in this study. Although the effect of TENS on some critical consequences such as breastfeeding, the interaction between mother and baby, NICU admission rate, and long-term infant complications have not been evaluated (14, 20).

In conclusion, this study has shown that the application of TENS at the first time of active labor phase can reduce at least two scores in patient labor pain with no complication neither in the mothers nor newborns. Future researches are needed to assess the application of TENS generalized to the whole population and evaluate the most effective dose of it. The strength of this study was to compare TENS with current pharmacological pain relief methods, instead of the non-intervention group. This study had some limitations; first, we could not match the groups for probable confounding factor such as anatomical, physiological, cultural, social factors, and anxiety and depression levels that might influence women’s pain level. The others were the small sample size, single-centered, and non-blinded intervention. 
